# Novel Insights into the Pathogenesis of Human Post-Primary Tuberculosis from Archival Material of the Pre-Antibiotic Era, 1931–1947

**DOI:** 10.3390/pathogens12121426

**Published:** 2023-12-07

**Authors:** Syeda Mariam Riaz, Kurt Hanevik, Lars Helgeland, Lisbet Sviland, Robert L. Hunter, Tehmina Mustafa

**Affiliations:** 1 Centre for International Health, Department of Global Public Health and Primary Care, Faculty of Medicine and Dentistry, University of Bergen, 5007 Bergen, Norway; tehmina.mustafa@uib.no; 2 Department of Clinical Science, Faculty of Medicine and Dentistry, University of Bergen, 5007 Bergen, Norway; kurt.hanevik@uib.no; 3 National Centre for Tropical Infectious Diseases, Medical Department, Haukeland University Hospital, 5021 Bergen, Norway; 4 Department of Pathology, Haukeland University Hospital, 5021 Bergen, Norway; lars.helgeland@uib.no (L.H.); lisbet.sviland@helse-bergen.no (L.S.); 5 Department of Clinical Medicine, Faculty of Medicine and Dentistry, University of Bergen, 5007 Bergen, Norway; 6 Department of Pathology and Laboratory Medicine, University of Texas Health Sciences Centre at Houston, Houston, TX 77030, USA; robert.l.hunter@uth.tmc.edu; 7 Department of Thoracic Medicine, Haukeland University Hospital, 5021 Bergen, Norway

**Keywords:** primary tuberculosis, post-primary tuberculosis, tuberculosis pneumonia, cavity

## Abstract

Objectives: Primary and post-primary tuberculosis (TB) are distinct entities. The aim of this study was to study the histopathology of primary and post-primary TB by using the unique human autopsy material from the pre-antibiotic era, 1931–1947. Material and Methods: Autopsy data were collected from the autopsy journals, and the human tissue was collected from the pathology archives at the Department of Pathology, the Gades Institute. Results: Histological presentations of TB lesions showed great diversity within a single lung. Post-primary TB starts as a pneumonia forming early lesions, characterized by the infiltration of foamy macrophages containing mycobacterial antigens within alveoli, and progressing to necrotic pneumonias with an increasing density of mycobacterial antigens in the lesions. These necrotic pneumonic lesions appeared to either resolve as fibrocaseous lesions or lead to cavitation. The typical granulomatous inflammation, the hallmark of TB lesions, appeared later in the post-primary TB and surrounded the pneumonic lesions. These post-primary granulomas contained lesser mycobacterial antigens as compared to necrotic pneumonia. Conclusions: Immunopathogenesis of post-primary TB is different from primary TB and starts as pneumonia. The early lesions of post-primary TB may progress or regress, holding the key to understanding how a host can develop the disease despite an effective TB immunity.

## 1. Introduction

Primary tuberculosis (TB) and post-primary TB are two distinct disease entities caused by the same organism, *Mycobacterium tuberculosis* (MTB) [1,2]. The evidence for these two disease entities dates to the early 1800s when TB disease was common and human autopsy material was easily available [3,4,5]. Primary TB occurs when an individual is infected with MTB for the first time without prior immunity to the infection [6]. Post-primary TB, as its name suggests, typically begins only after primary MTB infection has established systemic immunity [7,8]. The 10 million clinical TB cases diagnosed every year are mainly post-primary TB. Nevertheless, most knowledge on the pathogenesis of TB is restricted to primary TB, as this is possible to investigate by using animal models [9]. Post-primary TB develops fully only in humans, and no animal models can mimic the natural course of post-primary TB. How MTB causes its post-primary disease manifestation in an immune human host is therefore not well understood. Understanding the pathogenesis and immune processes responsible for the development of post-primary TB is an essential prerequisite for developing more effective therapies against this most common manifestation of TB.

Human material is needed to understand the immune pathogenesis of post-primary TB. However, the natural course of TB disease is altered by antibiotics, such that early lesions of post-primary TB may disappear, leaving only an apical scar [1,4,5]. It is, therefore, very difficult to obtain representative material to study the pathogenesis of this form of TB. Tissue samples from the pre-antibiotic era present a potential alternative, but these have not been stored systematically in most institutions. However, there are some exceptions, and in this study, we used human TB tissue from the pre-antibiotic era obtained from a Norwegian histopathological archive. Even though Norway has one of the lowest rates of TB today, TB was endemic in Norway until the 1950s [10]. The death rate from TB reached a peak around 1900, with 309 deaths per 100,000 inhabitants. In the year 1900, Norway passed a law that made medical treatment and registration of every TB infection and death mandatory [11]. Several institutions, such as Sanatoria, TB homes, and coastal hospitals, were established for the treatment of TB patients. Autopsies were performed on most of the cases dying at the hospitals. Detailed epidemiological, medical, and autopsy records were kept. The tissues were fixed in formalin, embedded in paraffin, and archived systematically. Such material contains corresponding epidemiological and medical histories and constitutes a resource that is not easily available around the globe.

This study aimed to understand the pathology of human post-primary TB by describing microscopic features of TB lesions representing the whole spectrum of disease development in humans and their correlations with the characteristics of patients, which included age, gender, and cause of death.

## 2. Material and Methods

**Figure 1:**
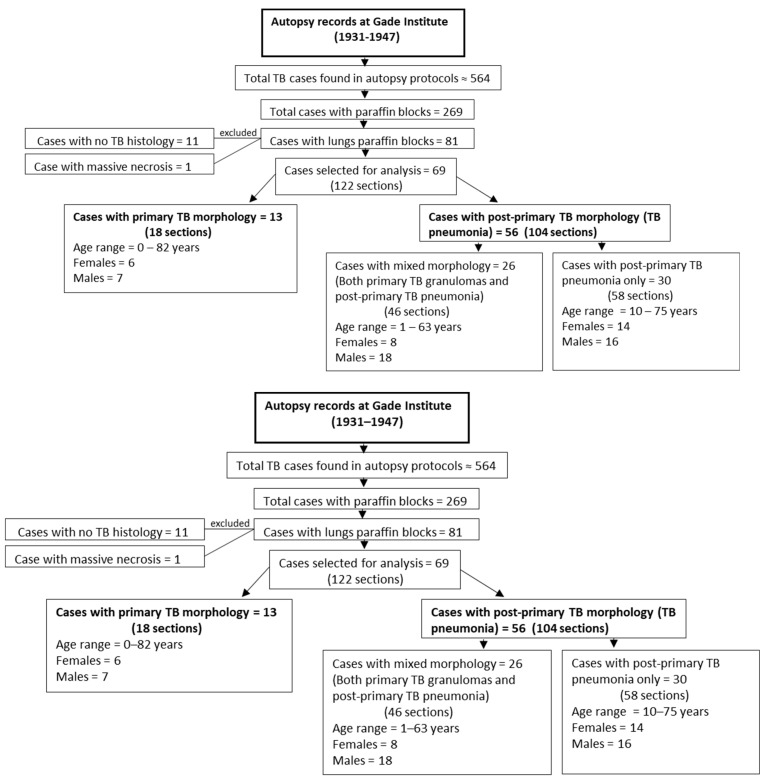
Flowchart showing case selection, age, and gender distribution for primary tuberculosis and post-primary tuberculosis groups examined in this study.

The tissue for this study was collected from the pathology archives at the Department of Pathology, the Gades Institute, Haukeland University Hospital, Bergen, Norway. Autopsies were carried out within 24 h after death. During the period selected for the study, from 1931–1947, tissues were processed according to a timely standard. That is, tissue specimens were submersed in 4% aqueous formaldehyde solution for several days before being embedded in paraffin, which was performed manually. Formalin-fixed paraffin-embedded blocks were then kept for long-term storage in the cellar at a cool temperature and normal humidity. The cadavers were stored before autopsy in the same building at a stable temperature of 5–8 °C and, from about 1940, with refrigeration at 4 °C [12]. Autopsy protocols from the years 1931 to 1947 were searched for TB patients. A total of 269 TB cases out of 564 TB cases found in autopsy protocols had available paraffin blocks. Among these 269 cases, 81 TB cases had lung paraffin blocks available, which were used in the microscopy study (Figure 1).

### 2.1. Histopathology Data

Formalin-fixed paraffin-embedded blocks were re-embedded and sectioned. Stainings for hematoxylin and eosin, acid-fast bacilli (AFB), and connective tissue were performed at the routine histology laboratory at the Department of Pathology, Haukeland University Hospital, Bergen, Norway. Masson’s trichrome staining was used for visualizing collagen fibers, and reticulum II staining was used for visualizing reticulin fibers. Masson’s trichrome staining, reticulin staining, and Ziehl Neelsen staining for AFB were performed in a VENTANA BenchMark Special Stains machine. A positive control for AFB was included in all batches. Stained sections for AFB and the MPT46 antigen were then scanned using a Hamamatsu NanoZoomer XR scanner (Hamamatsu, Japan) at 40×.

### 2.2. Immunohistochemistry

Immunohistochemistry for MTB antigens and secretory antigen MPT46 was performed manually using in-house rabbit polyclonal primary antibody [13,14]. Following deparaffinization and rehydration, sections were boiled for 20 min in citrate buffer (pH 6) in a microwave oven for antigen retrieval. Tris-buffered saline was used for washing between each incubation step. Peroxidase block (Dako Denmark A/S, Glostrup, Denmark) was applied for 20 min to block the activity of endogenous peroxidase, followed by serum-free protein block (Dako Denmark A/S) for 20 min to prevent non-specific binding. Primary antibodies were kept for overnight incubation at 4 °C for 22 h, followed by secondary antibody (labeled polymer HRP anti-rabbit) incubation for 40 min at room temperature. EnVision FLEX HRP Magenta Substrate Chromogen (Dako Denmark A/S) was used for visualization.

### 2.3. Quantification of AFB and MTB Antigens

For the quantification of AFB and MTB antigens, representative lesions based on good morphology for each lesion type containing AFB were selected. There was a deliberate selection bias towards lesions with clear and distinct morphologies. Some cases would have many representative lesions (ranging from 1 to 16), while other cases would have very few or none. For the quantification of AFB, Aperio ImageScope 12.3.3 was used to detect AFB in digitalized images. AFB were counted visually by human eye in the lesions after running a deconvolution algorithm. For MTB antigens quantification, the same cases selected for AFB were analyzed, and analysis was performed using QuPath (0.4.0) [15]. For intra-alveolar lesions like early lesions and early necrotic lesions, accumulations of macrophages within the alveoli were marked for analysis. For necrosis, all necrotic areas were not included in analysis. Only the areas showing the AFB or MTB antigen were annotated. For granulomas, whole granulomas, including necrosis and external rim, were annotated. The number of AFB counted and the area positive for MTB antigens were then divided by the area of the lesion analyzed to obtain measures of AFB and MTB densities.

### 2.4. Statistical Analysis

SPSS version 28 was used for all statistical data analyses. Pearson’s chi-squared test and *t*-test were used for group comparisons.

## 3. Results

Of the 81 cases with lung tissue available, 11 cases showed no TB histology, while one case had massive necrosis. Among the remaining 69 cases, 13 cases had only primary TB morphology, and 56 cases had post-primary TB pneumonia, with 26 of these exhibiting both primary granuloma lesions and post-primary TB pneumonia lesions (Figure 1).

All lesions reported in this study were confirmed as TB by the presence of MTB antigens and/or AFB in the lesions. The histological presentations showed great diversity within a single lung, with patterns ranging from typical primary TB granulomas to pneumonias, along with a varied extent of necrosis, cavitation, and fibrosis.

### 3.1. Primary TB

**Figure 2:**
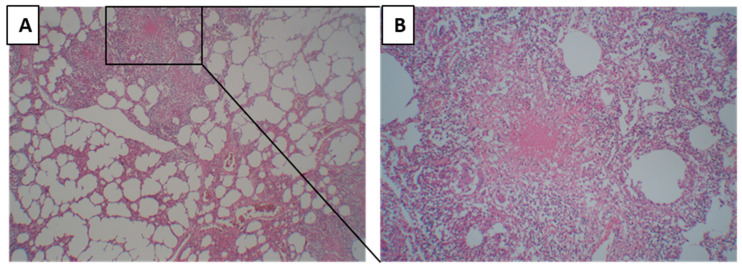
Morphology of primary tuberculosis. (**A**) Primary granuloma with the surrounding tissue where alveoli are being empty, and no pneumonia lesions are present. (**B**) Primary tuberculosis granuloma at higher magnification, characterized by homogenous necrosis surrounded by a rim of epithelioid cells and multinucleated giant cells, lymphocytes, and a fibrous capsule.

A lesion was defined as a primary granuloma when homogenous necrosis or fibrosis was surrounded by a rim of epithelioid cells and multinucleated giant cells, lymphocytes, and a fibrous capsule (Figure 2A,B). When there was no pneumonia in the surrounding tissue of the primary granuloma, it was grouped as primary TB.

Among the study population, there were four infants. Three of them had only primary TB granulomas in the histopathology without any stages of pneumonic infiltrations, indicating that this type of granuloma morphology is consistent with primary TB. The fourth infant did not have granulomas but had massive necrosis in the lungs with a very high bacterial load, and the mother died of miliary TB one month after birth. The infant was in close contact with the mother due to breastfeeding and had a fever almost since birth. He was tuberculin negative and died at 2 months of age, indicating that the infant was exposed to MTB from birth, and its immature immune system led to a histopathological manifestation of massive necrosis and no granuloma formation.

### 3.2. Post-Primary TB

**Figure 3:**
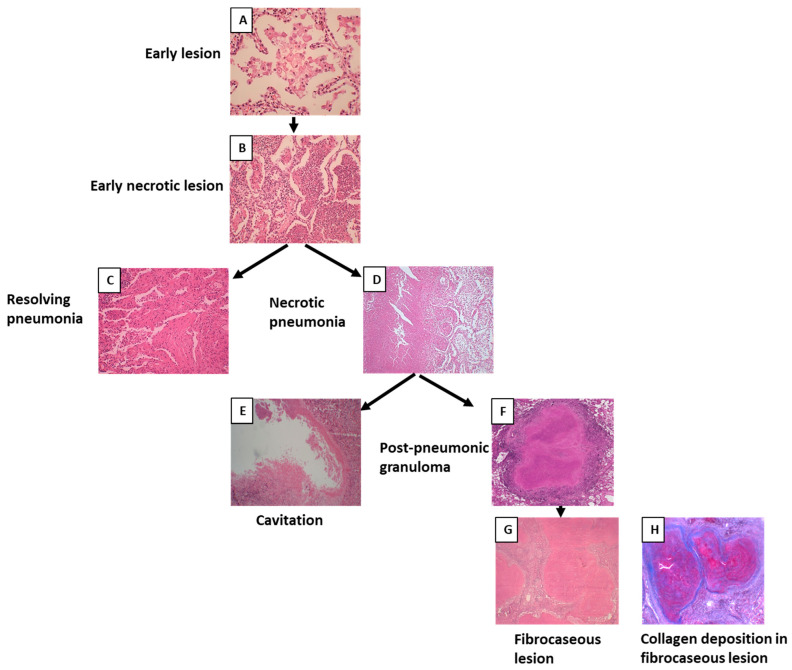
Morphology of post-primary tuberculosis. (**A**) Early lesion of post-primary tuberculosis where the accumulation of foamy macrophages can be seen within the alveoli. (**B**) Early necrotic lesion where lymphocytes infiltrated the alveolar space and necrosis started. (**C**) Resolving intra-alveolar lesion. (**D**) Areas of early necrotic lesions coalescing to form necrotic pneumonia. (**E**) Cavity formed after necrotic pneumonia sloughed off. (**F**) Post-pneumonic granuloma where necrosis was enclosed by fibrosis. (**G**) Fibrocaseous lesion showing fibrous deposition. (**H**) Masson’s trichrome staining showing collagen deposition in the fibrocaseous lesion.

Among 69 cases, 56 cases showed the morphology of post-primary TB with different stages of pneumonia. When pneumonia was seen in the lungs, the lesion was grouped as post-primary TB. Pneumonia in different stages of development was extrapolated from the histological patterns. The earliest lesion of tuberculous pneumonia was characterized by the accumulation of monocytes or macrophages in alveoli. The surrounding alveolar interstitium was infiltrated with lymphocytes, whereas no lymphocytes were present within the alveolar spaces. These lesions were small and were labeled as early lesions (Figure 3A). Early lesions were seen in 35 cases. Accumulations of lymphocytes and neutrophils were seen among the macrophages or monocytes within the alveolar spaces, and early necroses of accumulated macrophages and monocytes were observed. These lesions were considered the next-stage lesion, labeled as an early necrotic lesion (Figure 3B), and were seen in 30 cases. In three cases, intra-alveolar lesions were seen to be undergoing fibrosis and were labeled as resolving pneumonia (Figure 3C). In 17 cases, these early necrotic lesions seemed to coalesce to form larger areas of necrosis and were labeled as necrotic pneumonia (Figure 3D). Areas of necrosis in necrotic pneumonia in 16 cases became fissured and fragmented and seemed to be shedding off to form cavities (Figure 3E). In 16 cases, necrotic pneumonia was found to be enclosed by fibrosis, epithelioid cells, giant cells, and lymphocytes, forming a post-pneumonic granuloma (Figure 3F). In three cases, the center of the post-pneumonic granuloma was seen undergoing reticulin and collagen deposition and were termed as fibrocaseous lesions (Figure 3G,H).

### 3.3. Differences between Primary Granulomas and Post-Pneumonic Granulomas

**Figure 4:**
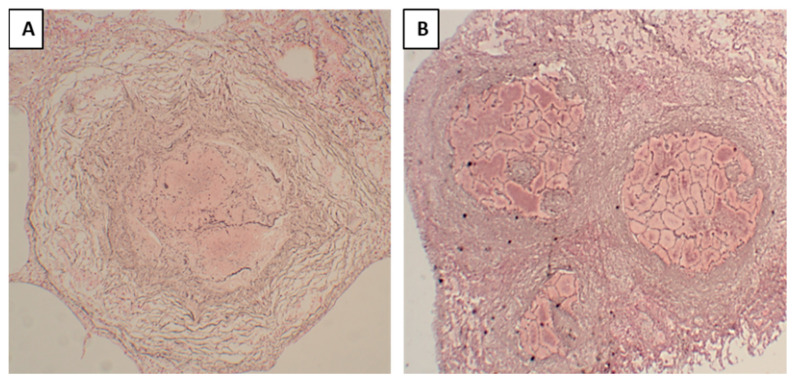
Reticular staining of post-pneumonic granulomas and primary tuberculosis granulomas, where reticulin fibers were stained black. (**A**). In the primary granuloma, the reticular outline of the alveolar septa was not visible within the necrotic area. (**B**). In the post-pneumonic granuloma, reticular staining showed a visible outline of the alveolar septal walls within the necrotic areas.

In primary granulomas, the reticular outline of the alveolar septa was not visible within the necrotic area (Figure 4A), indicating that this granuloma formation began with the accumulation of macrophages and lymphocytes mainly in the interstitium; the granulomas were formed first, and necrosis developed later. On the contrary, in post-pneumonic granulomas, reticular staining showed a visible outline of the alveolar septa (Figure 4B) within the necrotic areas, implying that these lesions started as pneumonia with the accumulation of macrophages within the alveoli, and then these lesions underwent early necrosis and later became confined by the granulomatous tissue. In fibrocaseous lesions, post-pneumonic granulomas were seen undergoing reticulin and collagen depositions (Figure 3H).

### 3.4. Correlation of Histopathology with Patient Characteristics and the Gross Pathology

Cases with post-primary lesions had more cavities (n = 30/56) (*p* = 0.04), more frequently died because of pulmonary TB (n = 20/56) (*p* = 0.04), and had less disseminated TB (n = 23/56) (0.02), compared to cases that only had primary granulomas. The cases with only primary granulomas more frequently showed the dissemination of TB to extrapulmonary organs (n = 10/13) (*p* = 0.02), and patients died more frequently (n = 8/13) (*p* = 0.001) because of disseminated TB, predominantly TB meningitis.

### 3.5. Acid-Fast Bacilli

**Figure 5:**
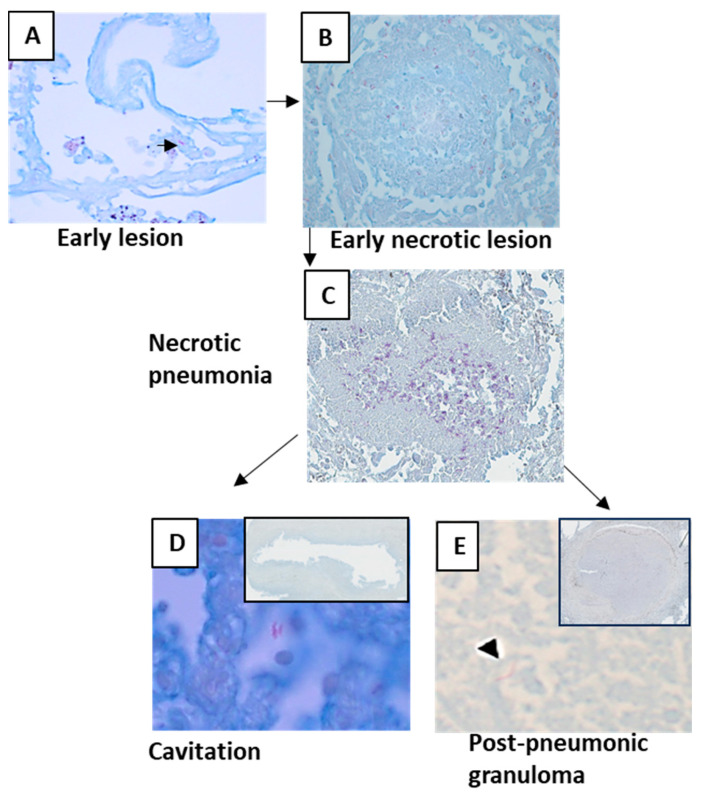
Acid-fast bacilli as seen in different lesions of post-primary tuberculosis. Densities of AFB varied in lesions. (**A**) AFB was present in a few early lesions. (**B**,**C**) AFB density increased with necrosis. (**D**) AFB density was lower in areas where cavities already formed. (**E**) Necrotic area of the post-pneumonic granuloma contained lesser AFB than the necrotic pneumonias.

Out of 69 cases examined, 30 cases were paucibacillary. They did not have any AFB in the whole tissue section, despite the positive staining of AFB controls. These cases were not included in the evaluation of AFB and MTB densities. Where AFB were seen, their densities varied across lesion types, as well as within the same case and between cases. AFB were frequently present in early lesions of cases with tuberculous pneumonia (mean density 416 AFB/mm^2^) (Table 1). Necrotic pneumonia lesions usually contained a higher density of AFB (mean density 557 AFB/mm^2^) (Figure 5) than the early lesions (*p* = 0.049), corroborating the association between high bacillary load and necrosis. However, few AFB (mean density 1.2 AFB/mm2) were found in the necrotic areas of post-pneumonic granulomas and in larger granulomas.

Compared with primary TB granulomas, the early lesion of post-primary TB contained 100 times more AFB.

Most early lesions are in the apices and regress spontaneously, leaving an apical scar. They have very few or no stainable AFB. In immunocompromised people, the lesions may contain many AFB and typically progress.

### 3.6. MTB Antigens

**Figure 6:**
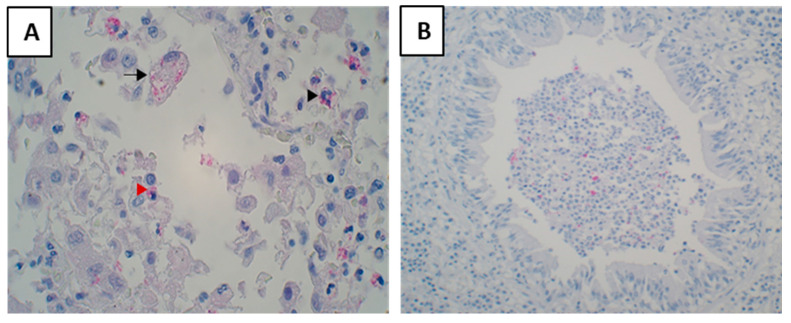
Staining of *Mycobacterial* antigens in tuberculosis lesions. They were found in macrophages, monocytes, and neutrophils (**A**). A lesion showing foamy macrophages (black arrow), monocytes (red arrowhead), and neutrophils (black arrowhead) containing antigens in the cytoplasm (**B**). An obstructed bronchi showing mycobacterial antigens in the cellular debris.

Antigens were seen as granular staining in the cytoplasm of foamy macrophages, monocytes and neutrophils were seen containing mycobacterial antigens (Figure 6A). Antigens were commonly found in cellular debris of obstructed bronchi (Figure 6B). Very rarely epithelial cells of bronchi were seen containing antigens.

**Figure 7:**
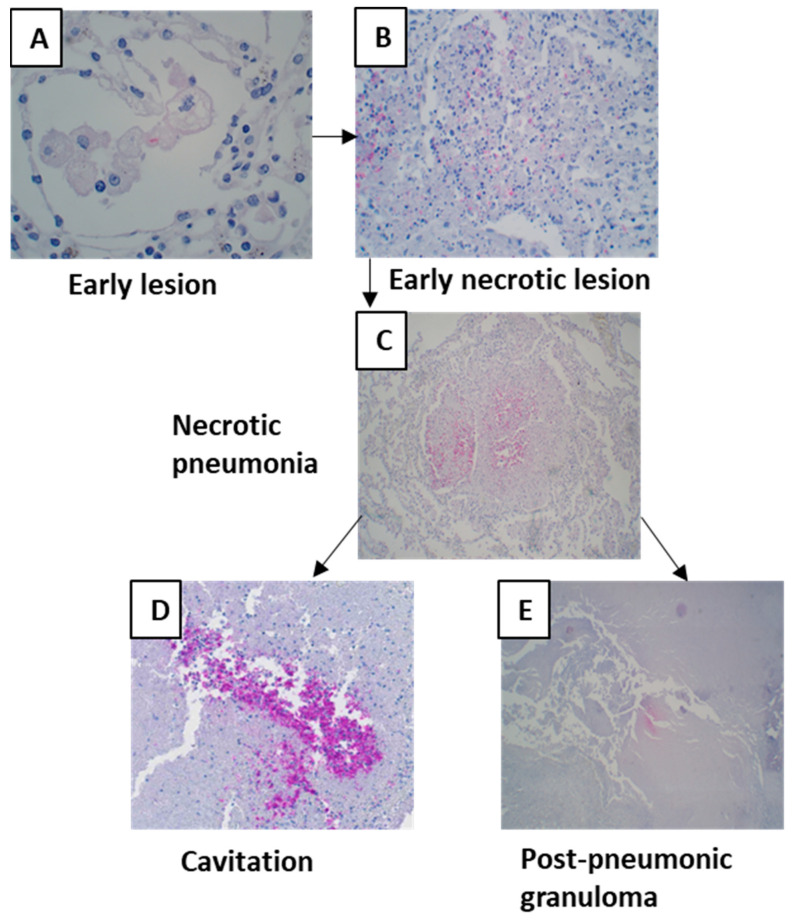
*Mycobacterial* antigen staining in different lesions of post-primary tuberculosis. (**A**) An early lesion containing *Mycobacterial* antigens. (**B**,**C**) *Mycobacterial* antigen densities increased with necrosis. (**D**) High densities of *Mycobacterial* antigens were in areas where tissue was sloughing off. (**E**) Necrosis of post-pneumonic granulomas also contained *Mycobacterial* antigens.

MTB antigens were not detectable in all lesions. Early lesions, except for very few cases, did not contain MTB antigens (Figure 7A). Compared with primary granulomas, the antigen density increased by five times in early lesions. Antigen staining density increased with necrosis (Table 1). Antigens could often be seen in necrosis where nuclear debris was still present (Figure 7C), and necrosis was not completely homogenous. The antigens were found more frequently in the largest quantity where tissue was sloughing off and a cavity was forming (Figure 7D).

## 4. Discussion

We studied the pathology of human lung TB lesions from the pre-antibiotic era to improve the understanding of post-primary TB. Correlating histology with the clinical history showed two distinct histopathological features for primary and post-primary TB. Even though pathologists from the pre-antibiotic era described distinct histopathologies for primary and post-primary TB [16,17], this has largely been ignored in contemporary thinking, where it is believed that primary TB granulomas are the only characteristic lesions seen in TB. The early lesions are seldom seen because there is no medical reason to biopsy a pneumonia that resolves with therapy. In our study, both TB pneumonia in various stages of development and granulomas could be found in biopsies from the same lung. Studies from the pre-antibiotic era described these two different histological patterns of TB using different nomenclatures, such as productive lesions for primary granulomas and exudative lesions for tuberculous pneumonia [4,5,16,18]. Productive lesions were associated with primary TB or childhood TB, and exudative lesions were seen in post-primary or adult-type TB. Our results show that primary granulomas were present more frequently in patients with extrapulmonary dissemination, increased TB mortality, and less cavitation. These are well-known characteristics for primary TB. Regarding post-primary TB, our results are in line with previous studies showing that post-primary TB pneumonia is associated with lesser extrapulmonary dissemination, lesser mortality, and more cavitation, characteristics associated with post-primary TB [17,19].

How TB starts depends on the immune status of an individual [20]. Based on our observation of the TB pathology in infants, we propose that primary infection TB starts as typical TB granulomas, as seen in animal models. The infection begins when the inhaled MTB is phagocytosed by the alveolar macrophages, and activation of MTB-specific adaptive immunity results in the accumulation of macrophages and lymphocytes at the site of infection, forming an early granuloma, mainly in the interstitium. Delayed-type hypersensitivity reaction results in the necrosis of immune cells, morphologically seen as caseous necrosis in the center of granulomas, mounting an effective immunity, which controls primary infection in more than 90% of the infected individuals [9].

The reaction to MTB infection in an individual who has developed immunity against the primary infection is different [19,21]. Based on our study, we can suggest that the reaction to the post-primary infection in a TB-immunized host is pneumonia. Post-primary TB starts as an early lesion of pneumonia. In the early lesions, macrophages in alveoli accumulate lipids and probably become a sanctuary for mycobacteria and evade host immunity. This is indirectly supported by our observation that early pneumonia lesions contained 500 times more AFB, compared to the primary TB granulomas. These lesions then undergo necrosis (early necrotic lesions). Early lesions progress to involve larger areas, causing necrotic pneumonia. The mycobacterial antigens begin to accumulate in these lesions, most probably a consequence of the degradation of AFB. These necrotic regions are either fragmented or expelled, forming a cavity or stabilizing as homogenous necrosis and are surrounded by granulomatous inflammation, forming post-pneumonic granulomas, which, in some cases, undergo fibrosis. Post-necrotic granulomas with fibrosis seem to indicate control of the infection, as these granulomas have lesser amounts of AFB and mycobacterial antigens. These post-pneumonic granulomas show the reticulin outline of alveolar septa, indicating that the lesion started within alveoli and then progressed to form a granuloma. A finding that supports our hypothesis is the absence of any kind of tuberculous pneumonia lesions found in children less than one year of age. Children less than one year of age are naïve to the infection, and their tissue reaction would be the result of the first infection with TB, inducing the formation of granulomas and not starting as pneumonia.

In three cases, early necrotic lesions were observed as a resolving pneumonia. In the pre-antibiotic era, serial X-rays were performed to monitor disease progression. These observations suggested that the TB lesions could be absorbed, indicating the reversible nature of such lesions. Dunham et al. observed noticeable absorption in more than 12% of the TB cases with clinical improvement [19]. Later CT examination of TB lesions suggested that a complete disappearance of a TB lesion can occur if a lesion heals before necrosis has developed. Otherwise, lesions invariably caused various degrees of fibrosis in association with bronchiovascular distortion or emphysema [22]. Scars of such healed lesions have been found in apices of the lungs in most people born in the 19th and 20th centuries in Europe and the USA [18].

The characteristic pattern seen on CT scans for post-primary TB or reinfection TB is the “tree-in-bud sign”, as shown by Jung-Gi Im et al. [22]. They found that the earliest CT finding of TB lesions was a centrilobular nodule or branching linear structure 2–4 mm in diameter (tree-in-bud). The microscopic and radiographic examinations of these lesions showed them mainly in the airways, with caseous material in both bronchioles and alveolar ducts with no evidence of interstitial involvement. Contrary to the tree-in-bud appearance, the CT scan of a patient with hematogenous dissemination showed evenly distributed nodules of uniform size [19]. We suggest that the tree-in-bud lesions seen on the CT are the same early lesions we found during microscopy, thus indicating that post-primary or reactivation TB starts as an early lesion of foamy macrophages in the alveolar space, spreading through bronchioles rather than through an expansion of pre-existing granulomas. These early lesions are reversible, and a better understanding of this spontaneous regression is the likely key to effective vaccine development.

A study performed by Mustafa et al. on human biopsies showed that major secreted MTB antigens were found in large numbers in pulmonary lesions that exhibited pneumonic infiltrates without typical TB granuloma formation [13]. Increased bacillary load and mycobacterial antigens accumulation in foamy macrophages could be associated with necrosis and cavity formation, as seen in murine pulmonary TB lesions [14]. In this study, we used secretory antigen MPT46, as this was found to be a highly secreted antigen in necrosis [14]. In our study MTB antigens increased with the progression of pneumonic lesions and the development of necrosis, suggesting a role for MTB antigens accumulation in tissue destruction and necrosis.

TB pathology of pneumonia rather than the primary granuloma was significantly associated with cavity formation, implying that the mechanism underlying cavitation starts with pneumonic infiltrations. The distinct immune processes involved in the development of caseous pneumonia and the formation of cavities are not known. Understanding these mechanisms can lead to the development of adjunct host-directed therapies to reduce the duration of TB treatment and organ damage.

The knowledge of tuberculosis learned by scientists in the pre-antibiotic era, where they described primary TB and post-primary TB as two distinct diseases with different clinical presentations, ages of onset, organ distributions of lesions, amounts of necrosis. and pathologies [4,16,17,18,19,20,21], has long been forgotten. Contemporary researchers look at primary granuloma as the characteristic lesion of tuberculosis and ignore tuberculous pneumonia as a TB lesion [23,24,25]. The prevailing understanding that primary granulomas are the key lesion of both primary and post-primary TB is an oversimplification of the disease processes, and it creates knowledge gaps and hinders the understanding of the true pathogenesis of post-primary TB required for the development of new vaccines and host-directed therapies. MTB is an obligate human parasite, and all the animal models are not natural hosts, as they cannot develop post-primary TB and transmit the disease as a human can [26]. Therefore, it implies that human material is needed to understand the immune pathogenesis of post-primary TB. Furthermore, any antibiotic treatment alters the host–pathogen interaction, interfering with the natural course of the disease process. In this study, we used untreated human material from the pre-antibiotic era, where the natural course of TB disease was not altered by antibiotics, and the early lesions of post-primary TB were preserved. This tissue material provided a unique opportunity to learn the pathogenesis of pulmonary TB and address several of the long-standing questions.

Our study has several limitations. There is a natural selection bias, as only people dying in hospitals were autopsied and included in the study. For some patients, the main TB representative area was not taken out on autopsy probably because TB was endemic, and TB diagnosis could easily be made upon gross examination. Nevertheless, TB is an unusual infection in that the whole spectrum of disease, including both early and late lesions, are frequently found in the same lung, thus making it possible to study a full-spectrum disease. Another limitation is that the cross-sectional study and the temporal relationship of lesions are not evident from the clinical information. However, it is possible to extrapolate the temporal relationship of the lesions based on biological plausibility and correlation using the description of the temporal progression of the lesions from a time when scientists had more possibility to study and correlate lesions seen on serial X-rays with the pathology seen on autopsies [5,19,21]. The study lacks detailed clinical data of patients; however, the clinical data available, such as age, gender, mortality, presence of cavities, and extrapulmonary tuberculosis were used to divide the morphologies of tuberculosis into primary and post-primary groups. The tuberculosis morphologies of four infants were taken as a reference for primary tuberculosis morphology.

## 5. Conclusions

Based on our observations, we conclude that TB pathology includes both TB granulomas and different stages of pneumonia. Lesions in different stages of development are found compartmentalized within the same lung. Post-primary TB is different from primary TB, and it starts in alveoli with the accumulation of macrophages. Understanding how these lesions progress or regress is important for the development of vaccines and immune therapy. Unfortunately, no animal models exhibit post primary TB lesions fully reflecting those in humans, and informative human tissues are seldom available today. Modern technologies make it possible to study formalin-fixed paraffin-embedded slides with a precision not possible a decade ago. Consequently, archival collections of tissues provide a unique opportunity to relearn the pathology of human TB, to formulate relevant questions and address them with new technologies, and to finally address several of the long-standing mysteries of the pathogenesis of TB.

## Figures and Tables

**Table 1:** Mean density of acid-fast bacilli (AFB) and ratio of the *Mycobacterium tuberculosis* (MTB) antigen-stained area in different types of lesions in cases where AFB were found.

Type of Lesion	Number of Cases	Number of Lesions	Mean Density of AFB per mm^2^	Mean Ratio of MTB Antigen-Stained Area per µm^2^
Primary TB Primary TB granuloma	8	11	4.1	0.1
Post-primary TB Early lesion of TB pneumonia	3	13	416	0.5
Early necrotic lesion of TB pneumonia	5	26	571	0.4
Necrotic TB pneumonia/necrosis	8	18	577	1.6
Cavitation	3	7	577	4.4
Post-pneumonic TB granuloma	4	5	1.2	0.05

## Data Availability

Data is unavailable due to privacy.

## Abbreviations

TB	Tuberculosis
MTB	Mycobacterium tuberculosis
AFB	Acid-fast bacilli
